# Moderate Fluid Shear Stress Could Regulate the Cytoskeleton of Nucleus Pulposus and Surrounding Inflammatory Mediators by Activating the FAK-MEK5-ERK5-cFos-AP1 Signaling Pathway

**DOI:** 10.1155/2018/9405738

**Published:** 2018-06-12

**Authors:** Dongping Ye, Weiguo Liang, Libing Dai, Yicun Yao

**Affiliations:** Guangzhou City Red Cross Hospital, The Fourth Affiliated Hospital of Medical College, Jinan University, Guangzhou 510220, China

## Abstract

We first applied moderate fluid shear stress to nucleus pulposus cells. The correlation of AP-1 with type II collagen, proteoglycan, Cytokeratin 8 protein, MAP-1, MAP-2, and MAP-4 and the correlation of AP-1 with IL-1*β*, TNF-*α*, IL-6, IL-8, MIP-1, MCP-1, and NO were detected. Our results document that moderate fluid shear stress could activate the FAK-MEK5-ERK5-cFos-AP1 signaling pathway. AP1 could downregulate the construct factors of cytoskeleton such as type II collagen, proteoglycan, Cytokeratin 8 protein, MAP-1, MAP-2, and MAP-4 in nucleus pulposus cell after the fluid shear stress was loaded. AP1 could upregulate the inflammatory factors such as IL-1*β*, TNF-*α*, IL-6, IL-8, MIP-1, MCP-1, and NO in nucleus pulposus cell after the fluid shear stress was loaded. Taken together, our data suggested that moderate fluid shear stress may play an important role in the cytoskeleton of nucleus pulposus and surrounding inflammatory mediators by activating the FAK-MEK5-ERK5-cFos-AP1 signaling pathway, thereby affecting cell degeneration.

## 1. Introduction

Degenerative disc disease is one of the major diseases currently affecting the quality of human life and aggravating the cost of social health care [1, 2]. The specific cause and pathogenesis of intervertebral disc degeneration have made great progress but have not yet been fully elucidated [3]. Many studies have suggested that intervertebral disc degeneration was caused by synergistic effects of various factors [4]. Stress environment, inflammatory mediator environment, genetic inheritance, and nutritional delivery disorder were considered to be strongly associated with intervertebral disc degeneration, and the stress environment was considered to be one of important reasons [5–7].

A recent study confirmed that compressive stress caused the change of cytoskeletal structure, function, and extracellular inflammatory mediators of nucleus pulposus through the changes in fluid shear stress inside the nucleus pulposus, thereby leading to intervertebral disc degeneration [8]. However, the mechanism by which fluid shear stress alters the cytoskeletal structure, function, and extracellular inflammatory mediators of nucleus pulposus remains unclear. Elucidating these issues is critical for the prevention and treatment of degenerative disc disease.

Cytoskeleton is the basis for maintaining complete cellular morphology and functional structure, including microtubules, microfilaments, and various cell organelles [9]. The main load-carrying components of the cytoskeleton are intermediate filaments and microtubules. Intermediate filament of nucleus pulposus cells is composed of type II collagen, proteoglycan, and Cytokeratin 8 protein [10]. The viscoelasticity is mainly determined by Cytokeratin 8 (keratin 8) [10]. Microtubule components, microtubule-associated proteins (MAPs) MAP-1, MAP-2, and MAP-4, could regulate cell morphology and redistribute cytoskeleton [10].

Our previous study found that the changes of cytoskeleton in nucleus pulposus were correlated with the intensity and action time of fluid shear stress. The cytoskeleton of nucleus pulposus could be reorganized and remodelled when the fluid shear stress altered in a certain range of intensity and action time. Cytoskeleton rupture, collapse, and cell death occurred when the intensity and action time of fluid shear stress exceeded a certain range. Moderate fluid shear stress activated the FAK-MEK5-ERK5-cFos-AP1 signaling pathway.

Activator protein 1 (AP-1) is a dimer formed by the interaction between members of the c-fos protein family and the c-jun protein family and is an important transcription factor in cells [11]. Many genes have AP-1 binding sites [11]. AP-1 regulates a series of pathophysiological processes, such as cell growth and differentiation, by binding to promoters or enhancers in genes [12]. AP-1 exists in many kinds of cells, and can be induced by stress, hormones, growth factors, cytokines, nerve media, heat shock, electroshock, ultraviolet ray, and oxygen stress [13]. Activated AP-1 regulates type II collagen, proteoglycan, Cytokeratin 8 protein, MAP-1, MAP-2, and MAP-4 by activating collagenase and matrix metalloproteinases (MMPs), thereby regulating cytoskeletal reorganization and remodeling [13]. Furthermore, activated AP-1 binds to AP-1 sites in promotors in interleukin (IL)-1*β*, tumor necrosis factor (TNF)-*α*, IL-6, IL-8, MIP-1, monocyte chemoattractant protein-1 (MCP-1), and nitric oxide (NO) genes and regulates their expression [13]. IL-1*β*, TNF-*α*, IL-6, IL-8, MIP-1, MCP-1, and NO are important inflammatory mediators in the degeneration of nucleus pulposus cells, especially IL-1*β* and TNF-*α* [14].

We hypothesized that moderate fluid shear stress could regulate the cytoskeleton of nucleus pulposus and surrounding inflammatory mediators by activating the FAK-MEK5-ERK5-cFos-AP1 signaling pathway, thereby affecting cell degeneration.

To verify this hypothesis, we applied moderate fluid shear stress to nucleus pulposus cells. The correlation of AP-1 with type II collagen, proteoglycan, Cytokeratin 8 protein, MAP-1, MAP-2, and MAP-4 and the correlation of AP-1 with IL-1*β*, TNF-*α*, IL-6, IL-8, MIP-1, MCP-1, and NO were detected.

## 2. Methods

**Figure 1:**
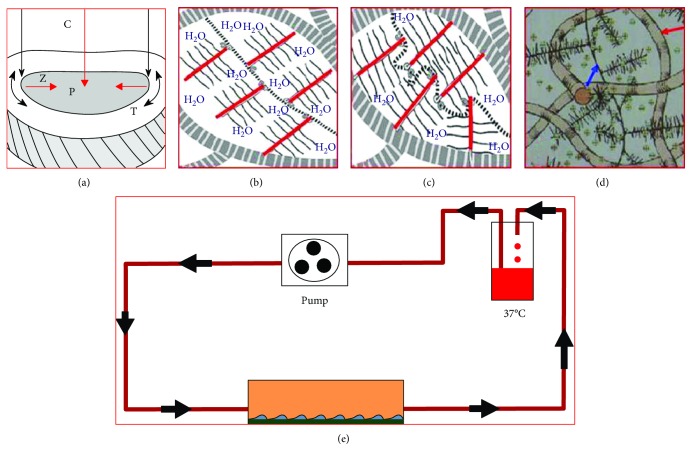
(a) The structure and function of intervertebral disc: compressive stress (C) produced hydrostatic pressure (P) in nucleus pulposus, then transferred the stress to the fibrous ring, and induced formation of tensile stress (T) in the fibrous ring tissue, induced counterforce of nucleus pulposus by fibrous ring (Z). (b) The proteoglycan was wrapped in a network of collagen fibers (the coarse line of the peripheral zebra). Proteoglycan was composed of a central chain (part of the dotted line) formed by hyaluronic acid. The clustered proteoglycan composed of the core protein (red line) and the glucan sulfate (solid line) was attached to the central chain. (c) Proteoglycan was hydrophilic swelling until pressure balanced with the tensile tension in the collagen fibers, the compress stress on the disc would squeeze part of the water out of the disc, then increased concentration of proteoglycan and increased potential energy of expansion inhibited further compression of nucleus pulposus. Once the compress stress was disappeared, the water returned to the tissue, the new balance was achieved, and the formation of fluid shear stress happened. (d) The blue arrow repressed proteoglycan, and the red arrow repressed collagen fiber. (g) Schematic diagram of fluid shear force device.

Our previous study confirmed that the change of cytoskeleton in nucleus pulposus was correlated with the intensity and action time of fluid shear stress. The cytoskeleton of nucleus pulposus could be reorganized and remodelled when the fluid shear stress altered in a certain range of intensity and action time. Cytoskeleton rupture, collapse, and cell death occurred when the intensity and action time of fluid shear stress exceeded a certain range. When the fluid shear stress was 12 dyn·cm^−2^ and action time was 45 minutes, the cytoskeletal reorganization of nucleus pulposus was most active, which avoided cytoskeletal collapse and effectively activated FAK-MEK5-ERK5-cFos signaling pathway and AP-1 (Figure 1).

RNA isolation kits were purchased from TaKaRa (Tokyo, Japan), and plasmid DNA preparation kits, restriction enzymes, T4 DNA ligase, and Taq polymerase were obtained from MBI Company (Shanghai, China). Western blot kits were purchased from Abcam (Cambridge, MA, USA). ELISA kit was purchased from Abcam (Cambridge, MA, USA).

### 2.1. Normal NP Cells Obtained

An immortalized human NP cell line was established by the hTERT-transfected, which has an extended lifespan, retains phenotypic features similar to primary parent NP cells, and provides a suitable model for studying the biology of NP cells. The cells were cultured in a T25 tissue culture flask with 6 mL DMEM containing 10% fetal bovine serum at 37°C, saturated humidity, and 5% CO_2_ for 3 days. This study set two groups. In the control group, no fluid shear stress was given. In the experimental group, fluid shear stress was given. The nucleus pulposus cells received 12 dyn·cm^−2^ multidirectional pulsed fluid shear stress for 45 minutes.

### 2.2. RNA Isolation and Real-Time PCR

Total RNA was extracted from nucleus pulposus by the TRIZOL method, and the RNA concentration and purity were tested. RNA 1 *μ*g was reverse transcribed into cDNA with a reverse transcription kit. PCR amplification system was 20 *μ*L, containing 10 *μ*L 2 × SYBR Premix Ex Taq mixture, 0.2 *μ*mol/L primer, 2 *μ*L twofold diluted cDNA, and sterile distilled water. Samples were amplified in real-PCR system (Roche). All primers are listed as follows. The obtained Ct value was divided by the Ct value of GAPDH. The relative expression levels of mRNA in different groups were calculated by 2^−ΔΔ*C*t^ method. All primer sequences were listed in Table 1. We have checked for normal distribution of results before using a parametric test.

### 2.3. Western Blot Assay for Detecting Protein Expression

Total protein was digested and extracted with RIPA lysate in each group. Protein concentration was measured by bicinchoninic acid assay. Proteins (50 *μ*g per well) underwent sodium dodecyl sulfate-polyacrylamide gel electrophoresis and were electrically transferred onto the polyvinylidene fluoride membranes. The membranes were blocked with 5% bicinchoninic acid for 1 hour and incubated with primary antibody at 4°C overnight. The next day, the membranes were incubated with secondary antibody for 1 hour after rewarming. Subsequently, the membranes were completely washed and visualized by using enhanced chemiluminescence. The expression of each protein was determined. We used the ImageJ for the quantification analysis. The solution of the antibody was 1 : 500 dilution. We have checked for normal distribution of results before using a parametric test.

### 2.4. Detection of Collagenase and MMPs in Extracellular Fluid by ELISA Assay

The expression of collagenase and MMPs in extracellular fluid was determined by ELISA assays using an ELISA kit purchased from Abcam (Cambridge, MA, USA). The sample groups consisted of blank wells, standard wells, and detected sample wells. We have checked for normal distribution of results before using a parametric test.

### 2.5. Statistical Methods

All data were analyzed by using SPSS 19.0 software and expressed as the mean ± standard deviation. Intergroup difference was compared with independent samples *t*-test. Statistical significance was set to *α* = 0.05 (bilateral). We have checked for normal distribution of results before using a parametric test.

## 3. Results

**Figure 2:**
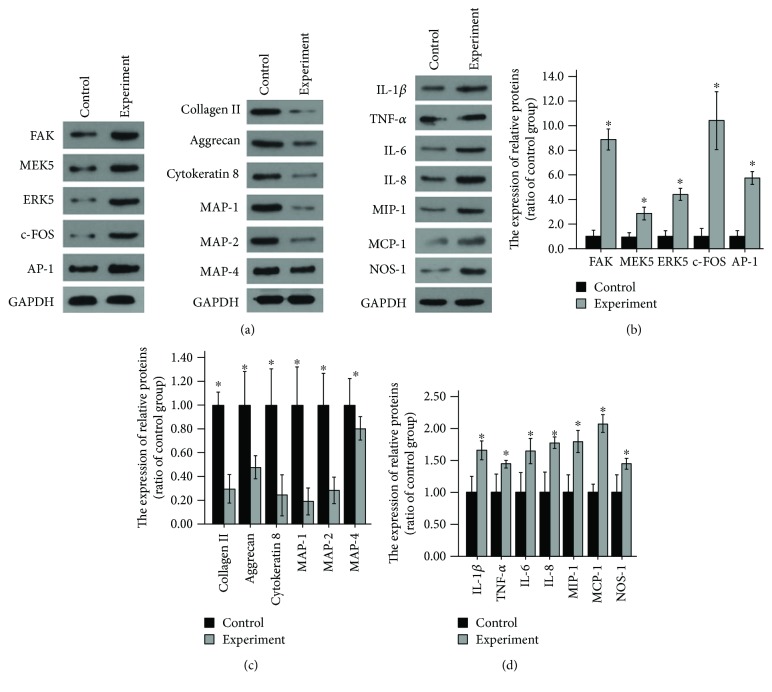
(a, b) FAK-MEK5-ERK5-cFos-AP1 pathway-related protein expression obviously increased compared with that before loading. (a, c) The expression of type II collagen, aggrecan, Cytokeratin 8, MAP-1, MAP-2, and MAP-4 proteins noticeably decreased compared with that before loading. (a, d) The expression of inflammatory mediators around nucleus pulposus cells dramatically increased, including IL-1*β*, TNF-*α*, IL-6, IL-8, MIP-1, MCP-1, and NO. ^∗^Repressed *p* value <0.05, statistical significance was set to *α* = 0.05 (bilateral). Experiments were repeated three times.

**Figure 3:**
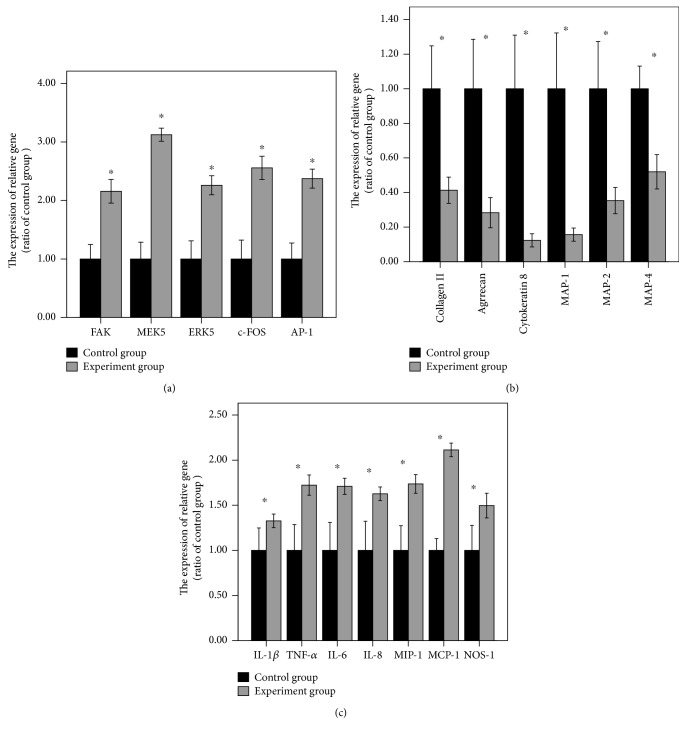
(a) FAK-MEK5-ERK5-cFos-AP1 pathway-related gene expression obviously increased compared with that before loading. (b) The expression of type II collagen, aggrecan, Cytokeratin 8, MAP-1, MAP-2 and MAP-4 mRNA noticeably decreased compared with that before loading. (c) The mRNA expression of inflammatory mediators around nucleus pulposus cells dramatically increased, including IL-1*β*, TNF-*α*, IL-6, IL-8, MIP-1, MCP-1 and NO. ^∗^Repressed *p* value < 0.05, statistical significance was set to *α* = 0.05 (bilateral). Experiments were repeated three times.

**Figure 4:**
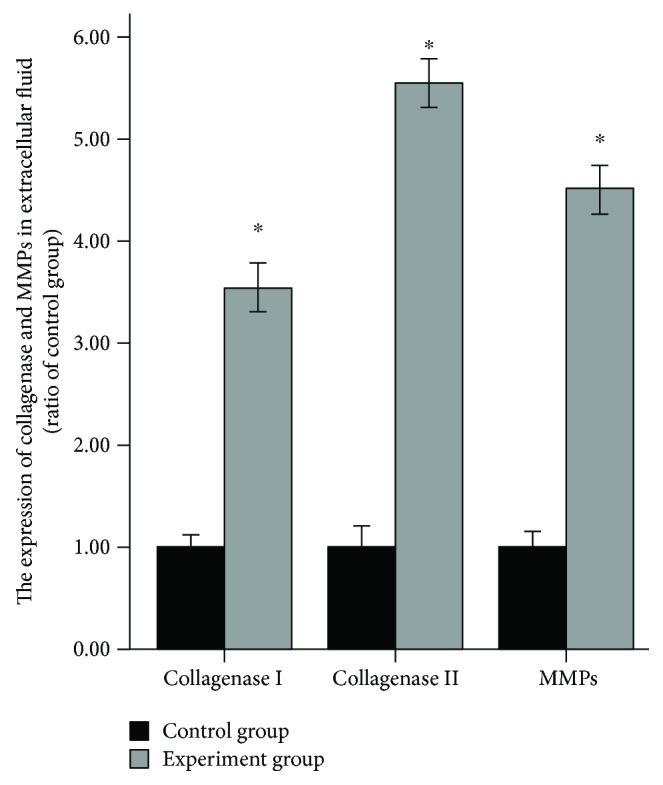
ELISA results revealed that types I and II collagenase and MMP expression remarkably increased compared with that before loading. ^∗^Repressed *p* value < 0.05; statistical significance was set to *α* = 0.05 (bilateral). Experiments were repeated three times.

1. After the loading fluid shear stress of 12 dyn·cm^−2^ for 45 minutes, FAK-MEK5-ERK5-cFos-AP1 pathway-related gene and protein expression obviously increased compared with that before loading. That was, this pathway was activated. There were significant statistical differences (Figures 2(a), 2(b), and 3(a)). Experiments were repeated three times.
2. After the loading fluid shear stress of 12 dyn·cm^−2^ for 45 minutes, ELISA results revealed that types I and II collagenase and MMP expression remarkably increased compared with that before loading. That was, AP-1 could promote the expression of types I and II collagenase and MMPs (Figure 4). Experiments were repeated three times.
3. After the loading fluid shear stress of 12 dyn·cm^−2^ for 45 minutes, RT-PCR and Western blot assay were used to measure the expression of type II collagen, aggrecan, Cytokeratin 8, MAP-1, MAP-2, and MAP-4. Results demonstrated that the mRNA and protein expression of type II collagen, aggrecan, Cytokeratin 8, MAP-1, MAP-2, and MAP-4 noticeably decreased compared with that before loading. That was, the expression of types I and II collagenase and MMPs increased, so cytoskeletal components were degraded and the expression decreased (Figures 2(a), 2(c), and 3(b)). Experiments were repeated three times.
4. After the loading fluid shear stress of 12 dyn·cm^−2^ for 45 minutes, the mRNA and protein expression of inflammatory mediators around nucleus pulposus cells dramatically increased, including IL-1*β*, TNF-*α*, IL-6, IL-8, MIP-1, MCP-1, and NO. That was, activated AP-1 bound to the AP-1 site of the promoter of the genes such as IL-1*β*, TNF-*α*, IL-6, IL-8, MIP-1, MCP-1, and NO, thereby increasing their expression, affecting the inflammatory mediators surrounding nucleus pulposus cells and causing degeneration of nucleus pulposus cells (Figures 2(a), 2(d), and 3(c)). Experiments were repeated three times.

## 4. Discussion

The c-Fos protein is composed of 380 amino acids with a molecular weight of 55 kD [15]. c-fos-encoded nuclear phosphoprotein plays an important role in the information transduction between external stimuli and transcription coupling, and it is also known as the “third messenger in the nucleus, immediate early gene.” c-Fos binds to c-Jun as a dimer, namely, AP-1 [15].

Numerous studies indicated that when osteoblasts were exposed to stretch, shearing force, supergravity and microgravity, mRNA, and protein expression of c-Fos was enhanced, and then the synthesis of AP-1 was promoted, which was possibly associated with the activation of MAPK pathway [16–18]. In 2002, Tolonen et al. first found high expression of c-Fos and c-jun in nucleus pulposus cells of patients with intervertebral disc herniation, which probably strongly associated with intervertebral disc degeneration [19]. In 2013, Yokoyama et al. first verified that MAPK pathway could activate c-Fos in nucleus pulposus cells [20]. Activated c-Fos could promote the degeneration of nucleus pulposus cells by inhibiting the expression of type II collagen and glycosaminoglycan in nucleus pulposus cells [20].

Our study suggested that nucleus pulposus cells increased mRNA and protein expression of c-Fos and then increased AP-1 expression at moderate fluid shear stress through activating FAK-MEK5-ERK5-cFos signaling pathway, which probably affected the changes of nucleus pulposus cytoskeleton and surrounding inflammatory mediators and influenced the degeneration of nucleus pulposus cells.

AP-1 is an important nuclear transcription factor, widely involved in cell growth, apoptosis, and regulation of inflammatory response [11]. The most classical form of AP-1 is a heterodimer composed of the Fos (c-Fos, Fos B) family and the Jun (c-Jun, Jun B) family and a homodimer composed of Jun family [19]. Extracellular stimulus signal could adjust the regulatory effect of downstream target genes on cell proliferation, differentiation, and apoptosis by activating AP-1 [19]. A previous study reported that AP-1 regulated inflammatory cell expression and controlled arthritis progression in articular chondrocytes [19]. AP-1 regulated the expression of MCP-1 in a variety of cells. In airway smooth muscle, AP-1 participated in MCP-1 expression and aggravated inflammatory response [19]. AP-1 likewise mediated MCP-1 expression in brain endothelial cells [11].

This study suggested that AP-1 regulated the cytoskeletal reorganization and remodeling through activating collagenase, and MMPs degraded type II collagen, proteoglycan, Cytokeratin 8 protein, MAP-1, MAP-2, and MAP-4. AP-1 regulated the environment of inflammatory mediators surrounding cells by increasing the expression of IL-1*β*, TNF-*α*, IL-6, IL-8, MIP-1, MCP-1, and NO. It is known that IL-1*β*, TNF-*α*, IL-6, IL-8, MIP-1, MCP-1, and NO are important inflammatory mediators in the degeneration of nucleus pulposus cells, and IL-1*β* and TNF-*α* are recognized as the main inflammatory mediators [21].

Chemokine is a class of small signal proteins secreted by cells, is chemotactic cytokine, and can control the directional migration of immune cells [22]. Yoshida et al. showed that from the nucleus pulposus to the outer layer of the annulus fibrosus, a zone of striated inflammatory granulation tissue is formed, and a large number of pain-causing inflammatory neurotransmitters and chemokines are formed and released, including TNF, prostaglandins, IL-1, IL-2, IL-6, IL-8, IL-10, NO, PLA2, MMPs, vasoactive polypeptide, and substance P [23].

MCP-1 is a typical member of the CC chemokine family, can recruit monocytes and cause inflammatory response [24]. Burke et al. removed normal and degenerative nucleus pulposus for culture and found that normal and degenerative nucleus pulposus cells could spontaneously produce MCP-1 and could recruit macrophages [25]. MCP-1 has been verified to be positively correlated with the degree of lumbar intervertebral disc herniation and the degree of pain [26]. The occurrence of MCP-1 can recruit inflammatory factors and further stimulate MCP-1 expression [26]. This cycle aggravates inflammatory cell infiltration and inflammatory response [26]. Therefore, MCP-1 may be the initiation factor of inflammatory response in intervertebral disc [26].

MMPs are involved in the degradation of the proteasome family of extracellular matrix in various tissues of the body [27]. Its main role is to regulate dynamic equilibrium of ECM, including various collagenases and elastases incorporated in the matrix and integrated in the plasma membrane [27]. The synthesis and decomposition of normal intervertebral disc matrix maintain a balance, and its decomposition is mainly through the generation and activation of catabolic enzymes, including MMPs and a disintegrin and metalloproteinase [28]. The degradation of the matrix is in equilibrium with the synthesis of the new matrix [28]. Bone morphogenetic protein-2 (BMP-2), insulin-like growth factor-1, BMP-7 (also known as osteogenic protein-1), growth differentiation factor 5, and transforming growth factor-*β* can stimulate the synthesis of matrix [29]. On the contrary, TNF-*α* and IL-1 inhibit the synthesis of extracellular matrix and improve their catabolism [29]. The balance of synthesis and degradation in the disc matrix determines the integrity of the intervertebral disc [29]. The imbalance of degradation and synthesis of matrix can induce the changes in the anatomical structure and functional characteristics of intervertebral disc [29]. If the catabolism is greater than the anabolism, the intervertebral disc matrix environment is disordered, and then the intervertebral disc affects degeneration [29].

TNF-*α* promotes the production of intervertebral disc cells [30]. The intervertebral disc cells can degrade proteoglycan and glycoprotein in matrix, such as fibronectin, laminin, and gelatin and can also degrade elastin and types II, III, IV, V, and VI collagen [30]. These functions can cause and accelerate the degeneration of intervertebral disc. It was found that TNF-*α* itself could promote the degradation of proteoglycan [30]. Furthermore, TNF-*α* can also participate in the metabolism of nucleus pulposus cells, lead to the decomposition of extracellular matrix, and play an important role in intervertebral disc degeneration and herniation [31].

IL-1 is mainly produced by macrophages and chondrocytes. IL-1 has various subtypes, such as IL-1*α* and IL-1*β* [32]. Nucleus pulposus cells are cartilage-like cells. There are IL-1 receptor (IL-1R) and IL-1R antagonist (IL-1Ra) in the nucleus pulposus cell membrane [32]. Normal disc tissue does not produce IL-1, but in degenerative intervertebral disc, nucleus pulposus cells produce a considerable amount of IL-1 [32]. A previous study verified that in degenerative and herniated intervertebral discs, the expression levels of IL-1*α* and IL-1*β* were correlated with the grade of intervertebral disc degeneration or the extent of intervertebral disc herniation [33]. In intervertebral disc cells, IL-1 stimulated the degradation of extracellular matrix (mainly proteoglycans and type II collagen) [34]. Furthermore, IL-1 also inhibited the synthesis of proteoglycan by inducing the expression of NO [34]. Kang et al. believed that NO expression was very low in the normal intervertebral disc, and IL-1 inhibited proteoglycan synthesis by inducing NO expression [35].

According to the results above, we speculated that moderate fluid shear stress may play an important role in the cytoskeleton of nucleus pulposus and surrounding inflammatory mediators by activating the FAK-MEK5-ERK5-cFos-AP1 signaling pathway, thereby affecting cell degeneration. Degenerated discs are frequently more painful when an individual is sitting, especially if he or she is slumped forward, putting more pressure on the lower back. Sitting upright in an ergonomic chair that provides low back support for the natural curve in the lumbar region can prevent irritating discs. Hanging a small mirror near their desk can allow patients to check posture and remind them to straighten up.

## 5. Conclusion

Our results may suggest a novel role for the FAK-MEK5-ERK5-cFos-AP1 pathway as an important modulator of human NP degeneration.

## Figures and Tables

**Table 1:** Primer sequences for PCR.

Primers	Forward	Reverse	Length
Collagen II	5′-TGGACGATCAGGCGAAACC-3′	5′-GCTGCGGATGCTCTCAATCT-3′	244 bp
Aggrecan	5′-ACTCTGGGTTTTCGTGACTCT-3′	5′-ACACTCAGCGAGTTGTCATGG-3′	80 bp
Cytokeratin 8	5′-CAGAAGTCCTACAAGGTGTCCA-3′	5′-CTCTGGTTGACCGTAACTGCG-3′	194 bp
MAP-1	5′-AACATGAGCGAGTTGGTCAAG-3′	5′-GCTCGTAGATGTCCGCGAT-3′	127 bp
MAP-2	5′-CTCAGCACCGCTAACAGAGG-3′	5′-CATTGGCGCTTCGGACAAG-3′	95 bp
MAP-4	5′-CCGGGCCAAAGTAGAGAAAAA-3′	5′-GACTGAATATGGCTGTAGCTCAC-3′	162 bp
IL-1*β*	5′-ATGATGGCTTATTACAGTGGCAA-3′	5′-GTCGGAGATTCGTAGCTGGA-3′	132 bp
TNF-*α*	5′-CCTCTCTCTAATCAGCCCTCTG-3′	5′-GAGGACCTGGGAGTAGATGAG-3′	220 bp
IL6	5′-ACTCACCTCTTCAGAACGAATTG-3′	5′-CCATCTTTGGAAGGTTCAGGTTG-3′	149 bp
IL8	5′-TTTTGCCAAGGAGTGCTAAAGA-3′	5′-AACCCTCTGCACCCAGTTTTC-3′	194 bp
MIP-1	5′-CTGTGCTGATCCCAGTGAATC-3′	5′-TCAGTTCAGTTCCAGGTCATACA-3′	61 bp
MCP-1	5′-CAGCCAGATGCAATCAATGCC-3′	5′-TGGAATCCTGAACCCACTTCT-3′	190 bp
NOS1	5′-TTCCCTCTCGCCAAAGAGTTT-3′	5′-AAGTGCTAGTGGTGTCGATCT-3′	118 bp
GAPDH	5′-GGAGCGAGATCCCTCCAAAAT-3′	5′-GGCTGTTGTCATACTTCTCATGG-3′	197 bp

## Data Availability

All relevant raw data will be freely available to any scientist wishing to use them for noncommercial purposes, without breaching participant confidentiality.
