# Corrigendum to “Moderate Fluid Shear Stress Could Regulate the Cytoskeleton of Nucleus Pulposus and Surrounding Inflammatory Mediators by Activating the FAK-MEK5-ERK5-cFos-AP1 Signaling Pathway”

**DOI:** 10.1155/2018/4597569

**Published:** 2018-09-27

**Authors:** Dongping Ye, Weiguo Liang, Libing Dai, Yicun Yao

**Affiliations:** Guangzhou City Red Cross Hospital, The Fourth Affiliated Hospital of Medical College, Jinan University, Guangzhou 510220, China

In the article titled “Moderate Fluid Shear Stress Could Regulate the Cytoskeleton of Nucleus Pulposus and Surrounding Inflammatory Mediators by Activating the FAK-MEK5-ERK5-cFos-AP1 Signaling Pathway” [1], a grant number was missing. The Acknowledgments section should be updated as follows:

This project was supported by the fund of Medical and Health Project of Guangzhou City (20151A011013, 20161A011013, 20171A011249, and 20171A011012), the fund of Guangzhou Municipal Science and Technology Bureau (201607010051), the fund of Guangdong Science and Technology Department (2013B021800070), and the Guangdong Provincial Natural Science Fund of China (2015A030313736 and 2018A030310021).
